# Feasibility of a Low-Fidelity Pediatric Simulation-Based Continuing Education Curriculum in Rural Alaska

**DOI:** 10.7759/cureus.8288

**Published:** 2020-05-26

**Authors:** Elizabeth Sanseau, Anita Thomas, Elizabeth Jacob-Files, Asela Calhoun, Susan Romero, Shruti Kant

**Affiliations:** 1 General Pediatrics: Emergency Medicine, Children's Hospital of Philadelphia, Philadelphia, USA; 2 Pediatrics, Seattle Children's Hospital, Seattle, USA; 3 Qualitative Health Research, University of Washington and Seattle Children’s Hospital, Seattle, USA; 4 Community Health Aide Program: Education, Yukon-Kuskokwim Health Corporation, Bethel, USA; 5 Pediatric Critical Care: Pediatric Simulation, Alaska Native Medical Center, Anchorage, USA; 6 Emergency Medicine: Pediatric Emergency Medicine, University of California, San Francisco, USA

**Keywords:** simulation-based medical education, curriculum planning, continuing professional development, pedagogical practice, feasibility assessment, community health aides and practitioners, native alaska, rural medicine, pediatric emergency medicine

## Abstract

Introduction

Simulation-based continuing education (SBCE) is a widely used tool to improve healthcare workforce performance. Healthcare providers working in geographically remote and resource-limited settings face many challenges, including the development and application of SBCE. Here, we describe the development, trial, and evaluation of an SBCE curriculum in an Alaska Native healthcare system with the aim to understand SBCE feasibility and specific limitations.

Methods

The perceived feasibility and efficacy of incorporating a low-fidelity medical simulation curriculum into this Native Alaskan healthcare system was evaluated by analyzing semi-structured interviews, focus groups, and surveys over a 15-month period (August 2018 - October 2019). Subjects were identified via both convenience and purposive sampling. Included were 40 healthcare workers who participated in the simulation curriculum, three local educators who were trained in and subsequently facilitated simulations, and seven institutional leaders identified as “key informants.” Data included surveys with the Likert scale and dichotomous positive or negative data, as well as a thematic analysis of the qualitative portion of participant survey responses, focus group interviews of educators, and semi-structured interviews of key informants. Based on these data, feasibility was assessed in four domains: acceptability, demand, practicality, and implementation.

Results

Stakeholders and participants had positive buy-in for SBCE, recognizing the potential to improve provider confidence, standardize medical care, and improve teamwork and communication, all factors identified to optimize patient safety. The strengths listed support feasibility in terms of acceptability and demand. A number of challenges in the realms of practicality and implementation were identified, including institutional buy-in, need for a program champion in a setting of staff high turnover, and practicalities of scheduling and accessing participants working in one system across a vast and remote geographic region. Participants perceived the simulations to be effective and feasible.

Conclusion

While simulation participants valued an SBCE program, institutional leaders and educators identified veritable obstacles to the practical implementation of a structured program. Given the inherent challenges of this setting, a traditional simulation curriculum is unlikely to be fully feasibly integrated. However, due to the overall demand and social acceptability expressed by the participants, innovative ways to deliver simulation should be developed, trialed, and evaluated in the future.

## Introduction

Simulation as a training tool is increasingly utilized in medical education, as it is demonstrated to be effective in improving patient safety and reducing health care costs [1-3]. The degree to which the simulation training reflects reality is described along a continuum of low- to high-fidelity, which can be adjusted according to the learning objectives of the simulation [4]. High-fidelity is not necessarily superior to low-fidelity to achieve educational learning objectives [5-6]. Barriers to incorporating high-fidelity simulation include insufficient availability, cost, lack of access and trained faculty, and time constraints [7]. While a low-fidelity simulation program is inexpensive and requires little to no additional equipment to implement, it does require at least the eight critical factors identified by the Joint Commission Journal on Quality and Patient Safety: science, staff, supplies, space, support, systems, success, and sustainability [8].

Geographic and ethnic disparities in healthcare service more heavily impact vulnerable populations of infants and children when compared to adults, and death rates for American Indian/Alaska Native (AI/AN) infants and children, the majority of those who reside in a rural area, are nearly three times higher than for Caucasians. Overall, the pediatric death rate for AI/AN youths to 19 years of age was 73.2 as compared with 29.1 for Caucasian youths from 1999-2009, with the AI/AN pediatric death rates highest for the Alaska region across all age categories (P<0.1) [9]. Influenza and pneumonia rank as the highest causes of mortality in Alaska [10].

The backbone of the healthcare delivery workforce in the Southwest Native Alaska villages is the Community Health Aide/Practitioner (CHA/P), with hospital personnel, including nurses, advanced practitioners, and physicians, staffing the sub-regional centers and regional hospital. The CHA/Ps practice broad-spectrum medicine, including primary and preventative, chronic, acute, and emergency services. They service 47 Alaska Native villages surrounding the regional city hub, with approximately 25,000 residents, spanning a river delta region more than 50,000 square miles in size [11-14]. The land consists of coastal wetlands, tundra, and mountains and is accessible off the traditional road system via plane, boat, dog sled, or snow machine, colloquially known as the “bush.” The approximately 170 CHA/Ps working in this region are often the sole source of healthcare in the village. They report to a supervising provider located in the sub-regional clinic or main hospital hub and must be prepared to handle any emergency medical situation that arises.

The CHA/Ps, nurses, and providers are required to complete continuing education (CE) as part of the maintenance of certification. Given the geographically remote and resource-limited setting of the hospital, this usually means traveling far, which is time and cost-intensive. CHA/Ps have access to CE boot camps in the regional hospital and until now, their simulation experience is limited to simple procedural task trainers and standardized certificate courses (e.g. Acute Life Support).

The inspiration for this project arose from a direct request for simulation-based education from experienced CHA/Ps working in a sub-regional clinic setting in Southwest Alaska. Given the unique job description of these providers, including specific medical knowledge, skills, and teamwork/communication capabilities required for success on the job, simulation-based education seems like a potentially suitable and beneficial endeavor [15]. The authors subsequently conducted an assessment to capture the specific needs of this community. Providers reported a lack of a formal simulation program and a desire to have one. Specifically, those surveyed requested scenarios for pediatric respiratory distress, likely in response to the disproportionately high rates of respiratory infections and pulmonary chronic disease in the region [16].

Given the declared need for an SBCE program for these providers working with this population with significant health disparities, the authors developed and piloted a low-fidelity simulation curriculum for pediatric respiratory distress scenarios common in the region [17]. The simulations were launched as part of this study developed with the aim to assess the feasibility of the curriculum in the domains of acceptability, demand, practicality, and implementation, in addition to the perceived efficacy. The feasibility study methodology was selected because community partnerships needed to be established and a paucity of prior studies exist that are relevant to the target population [18]. This study was evaluated in a real-world setting, recognizing the difficulties of conducting an internally validated, highly controlled efficacy trial in a community site. Local practitioners and community members were actively involved in meaningful ways, from program inception through design and execution.

## Materials and methods

Study design, participants, sample size, and sampling

Two CE simulation-based boot camps were held for CHA/Ps and one day of simulations was held for hospital-based nurses and emergency room (ER) technicians facilitated by outside simulation educators (ES, SR). Instructors were one pediatrician who worked at the local institution (ES) and one traveling pediatric critical care nurse practitioner (SR), each with over three years of simulation facilitation experience. A convenience sample of 11 CHA/Ps, 15 nurses, and two ER technicians participated in the sessions and were invited to submit survey responses evaluating their perception of the effectiveness and feasibility of the activity in the domains of acceptability, demand, practicality, and implementation. These sessions led by outside facilitators are grouped together as Survey Group #1.

In an effort to implement a sustainable program, we trained local educators of the hospital nursing staff and the CHA/Ps simulation facilitation and debriefing with a one-day workshop and subsequently asked them to facilitate two simulation boot camp days with a total of 12 CHA/P participants. All participants received official CE credit for their participation. These sessions facilitated by trained local educators are grouped together as Survey Group #2.

Of the five local educators who participated in the training, only three were able to subsequently facilitate the two simulation days due to competing work demands, with one of them facilitating both sessions. Following these simulation days, we conducted focus groups of the facilitators to explore their perceptions of the tool. This is captured as Focus Groups #1 and #2.

In addition, seven local “key informant” hospital leaders were identified via purposeful sampling to participate in semi-structured interviews guided with the intent to explore their perception of the feasibility of implementing a simulation program at their institution.

**Figure 1:**
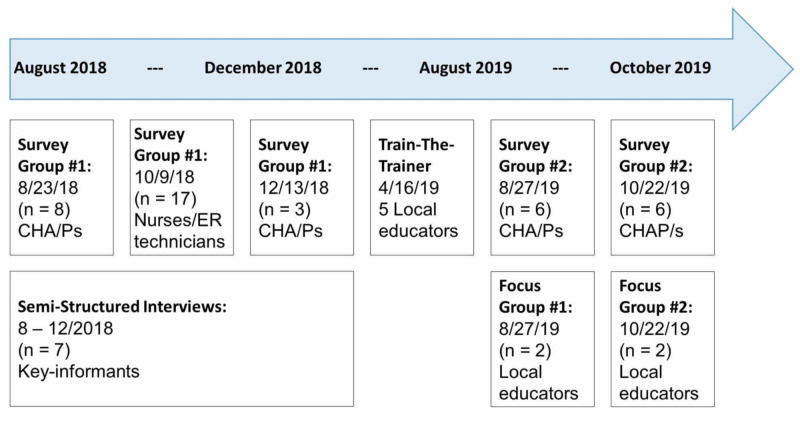
Timeline CHA/Ps: Community Health Aide/Practitioners; ER: Emergency Room

These data collection points are summarized in Figure 1.

Data collection & analysis

Simulation participants were surveyed on their perception of the effectiveness of the exercise on the basis of realism, usefulness in reviewing resuscitation and medical management skills, and whether the debrief felt safe and relevant, using the 5-point Likert scale (1 = strongly disagree, 3 = neutral, 5 = strongly agree) and the mean, standard deviation, median, and ranges were calculated [19]. See Table 1.

**Table 1:** Post-simulation questionnaire of participants

Likert Scale (1: Strongly disagree, 2: Disagree, 3: Neutral, 4: Agree, 5: Strongly agree)
This simulation is relevant to my work
This simulation was realistic
This simulation effectively taught me basic resuscitation skills
This simulation effectively taught me medical management skills
I felt the debrief was conducted in a safe environment
I felt the debrief promoted reflection and team discussion

Feasibility data were collected as dichotomous yes/no answers and qualitative responses to participant surveys (Table 2), as well as key informant, semi-structured interviews (Table 3) and educator focus group interviews (Table 4). The questions were framed to assess the four feasibility dimensions of acceptability, demand, practicality, and implementation. The content of the qualitative responses, interviews, and focus groups was analyzed. A realist thematic analysis approach was used to establish categories of concepts and themes, focusing on reporting the experiences, meanings, and realities across respondents [20]. The codebook, which contained mostly semantic themes, was developed directly from the text [21]. In the final round of coding, excerpts from each major theme were used to refine and define sub-themes in the context of the four feasibility domains listed above.

**Table 2:** Post-simulation participant survey CHA/Ps: Community Health Aide/Practitioners; ER: Emergency Room

What is your role?
CHA/P
Nurse
ER Technician
Other
ACCEPTABILITY:
Were you satisfied by the simulation experience today?
Yes / No
Would you be interested in participating in future simulations?
Yes / No
Do you think simulation is an appropriate teaching method for your level of training?
Yes / No
Would you like to see simulations as a regular occurrence where you work?
Yes / No
Do you think simulation should be required of all staff where you work?
Yes / No
If simulation became a regular part of training at your organization, do you think there would be an overall positive or negative effect on the organization? (circle one)
Positive / Negative
DEMAND:
Have you participated in simulation in the past?
Yes / No
If simulation were offered on a more regular basis at your institution, do you think it would help guide behaviors?
Yes / No
IMPLEMENTATION:
Did you think this simulation was worth your time?
Yes / No
Do you think participating in regular simulations where you work would be easy or difficult?
Easy / Difficult
PRACTICALITY:
Does your workplace currently have simulation equipment? (circle one)
Yes / No
If a simulation program were offered, would you be able to attend the simulations in the future?
Yes / No

**Table 3:** Key informant semi-structured interview guide CHA: Community Health Aide; CHA/Ps: Community Health Aide/Practitioners; CME: Continuing Medical Education; ER: Emergency Room; YKHC: Yukon-Kuskokwim Health Corporation

What is your role?
ACCEPTABILITY:
Is the concept of a new simulation program at YKHC attractive? (circle one)
Yes / No
Please explain:
Do you think simulation is an appropriate teaching method for this population? (circle one)
Yes / No
Please explain:
What might be some positive effects of implementing a simulation program?
What might be some negative effects of implementing a simulation program?
DEMAND:
Do you think there is a demand for simulation at YKHC? (circle one)
Yes / No
Please explain:
Do you think regular simulations with hospital staff / students / CHA’s would guide behaviors?
Yes / No
Please explain:
If a simulation program were developed for use at YKHC, would there be institutional buy-in to implement it?
Yes / No
If yes, how so? (i.e.: CME, etc.)?
IMPLEMENTATION:
Do you think implementing a simulation program would be easy or difficult?
If easy, please explain:
If difficult, please explain:
What would be barriers/limitations to implementing a structured simulation program? (i.e.: resources, community buy-in, etc.)
PRACTICALITY:
Do you think simulation would have a positive or negative effect on the participants? (circle one)
Positive / Negative
If positive, please explain:
If negative, please explain:
If a simulation program were developed for YKHC, do you think the program could be carried out and sustained without outside interventions moving forward?

ACCEPTABILITY:
Is the concept of a new simulation program at YKHC attractive? (circle one)
Yes / No
Please explain:
Do you think simulation is an appropriate teaching method for this population? (circle one)
Yes / No
Please explain:
What might be some positive effects of implementing a simulation program?
What might be some negative effects of implementing a simulation program?
DEMAND:
Do you think there is a demand for simulation at YKHC? (circle one)
Yes / No
Please explain:
Do you think regular simulations with hospital staff / students / CHA’s would guide behaviors?
Yes / No
Please explain:
If a simulation program were developed for use at YKHC, would there be institutional buy-in to implement it?
Yes / No
If yes, how so? (i.e.: CME, etc.)?
IMPLEMENTATION:
Do you think implementing a simulation program would be easy or difficult?
If easy, please explain:
If difficult, please explain:
What would be barriers/limitations to implementing a structured simulation program? (i.e.: resources, community buy-in, etc.)
PRACTICALITY:
Do you think simulation would have a positive or negative effect on the participants? (circle one)
Positive / Negative
If positive, please explain:
If negative, please explain:
If a simulation program were developed for YKHC, do you think the program could be carried out and sustained without outside interventions moving forward?

**Table 4:** Focus-group interview guide

What is your role?
Age
Gender
Race/ethnicity
Job title
Highest level of educational attainment
Where they spend most of their time (e.g. research, hospital, education)
What is your prior experience facilitation simulations, prior to this project?
How long have you been in clinical practice?
What is your training background?
Did you participate in the April workshop on simulation facilitation and debrief prior to facilitating today’s simulation sessions?
Overall, did you feel like the April simulation workshop was a good use of your time? Why or why not?
Do you feel that facilitating and debriefing today’s simulations was a good use of your time? Why or why not?
Do you feel that the trainees participating in your simulations today thought it was a good use of their time? Why or why not?
Do you feel that the April simulation workshop prepared you to facilitate the simulation and debriefing today? Why or why not?
Which aspects of the April simulation workshop training really resonated with you?
(What, if any, aspects from your teaching practice adjusted after the April simulation workshop? Which aspects of the training influenced this adjustment?
Do you think the April simulation workshop training built your confidence in your ability to facilitate simulation and debriefing today? If yes, how? What aspects of the workshop helped to build confidence?
Do you foresee yourself using simulation as a teaching tool moving forward? If yes, how? If no, why not?
Do you think you’ll use simulation as a teaching modality more now after the April workshop and this experience facilitating the simulations?
What would you improve about the April simulation workshop (content, logistics, or anything)? Or, what did not seem to “work” or resonate with you as an element that you could incorporate into your teaching practice?
What would you improve about the simulations that you ran today (content, logistics, or anything)? Or, what did not seem to “work” or resonate with you as an element that you could incorporate into your teaching practice?
Identify and explain any barriers you may face to incorporating simulation into your teaching practice, now and in the future?
How do think simulation affects the quality of your teaching?
How do you think using simulation as a teaching modality differs, if at all, from other teaching modalities you typically use?
Do you have any thoughts to share about how you foresee a simulation program fitting in with the direction or vision of how your institution is evolving?
Assuming you found the work compelling, what structure would be helpful for sustainment?
Anything else to add?

Acceptability was translated as the perception that the SBCE would be both desirable and culturally appropriate, with the mostly Alaska Native population that makes up the CHA/P workforce, the nursing staff, and educators in a hospital known to have a high staff turnover rate. Demand was operationalized as the perceived need for the SBCE. Practicality was linked to the costs (financial and temporal) associated with the program. Implementation was evaluated as the likelihood that the intervention could be fully integrated into the local CE coursework without needing ongoing outside resources.

This investigation was approved by the Seattle Children’s Hospital Institutional Review Board and the Yukon-Kuskokwim Health Corporation (YKHC) Human Subjects Committee.

## Results

Participants

Five local educators participated in a simulation facilitation workshop and three of the five subsequently led two simulation days. These three educators train CHA/Ps or nurses, average over 25 years of clinical nursing experience each, and have simulation experience in facilitating the Advanced Cardiac Life Support (ACLS) course. Of the 40 simulation participants, 23 were CHA/Ps, 15 nurses, and two ER technicians. Twelve of the CHA/Ps participated in simulations facilitated by the newly trained local educators (Survey Group #2) while the other 28 participants were led by external simulation facilitators (Survey Group #1). The seven key informants that participated in the semi-structured interviews included three physicians, two doctorate, and two nurse administrators; all informants were selectively identified due to their leadership roles in the hospital system, Community Health Aide Program, and nursing/provider education.

Effectiveness outcomes

All 40 simulation participants completed the post-simulation questions assessing feasibility and effectiveness. When asked to evaluate the effectiveness of the simulations on the Likert scale across six questions, the curriculum received a median of 5, mean of 4.59, standard deviation of 0.23, and range 3-5. When comparing the responses of participants with outside facilitators (Survey Group #1) vs local newly trained simulation facilitators (Survey Group #2), the mean (standard deviation) was only slightly higher (4.75 (0.19) vs 4.42 (0.14)) and the range of responses was respectively wider (3-5 vs 4-5) (Table 5).

**Table 5:** Participant feedback on simulation effectiveness CHA/P: Community Health Aide/Practitioners; ER: Emergency Room

(Likert Scale: 1 = Strongly disagree, 2 = Disagree, 3 = Neutral, 4 = Agree, 5 = Strongly agree)				
Statement	Participants	Mean (Standard Deviation)	Median	Range
This simulation is relevant to my work (Survey Group #1)	CHA/P (n=11)	4.74 (0.47)	5	4 – 5
	Nurse, ER technician (n=17)	4.94 (0.24)		
This simulation is relevant to my work (Survey Group #2)	CHA/P (n=12)	4.58 (0.51)	5	4 – 5
This simulation was realistic (Survey Group #1)	CHA/P	4.27 (0.79)	5	3 – 5
	Nurse, ER Technician	4.82 (0.39)		
This simulation was realistic Survey Group #2)	CHA/P	4.25 (0.62)	5	3-5
­This simulation effectively taught me basic resuscitation skills (Survey Group #1)	CHA/P	4.64 (0.50)	5	3 – 5
	Nurse, ER Technician	4.59 (0.71)		
­This simulation effectively taught me basic resuscitation skills (Survey Group #2)	CHA/P	4.33 (0.49)	4	4 – 5
This simulation effectively taught me medical management skills (Survey Group #1)	CHA/P	4.73 (0.47)	5	4 – 5
	Nurse, ER Technician	4.82 (0.39)		
This simulation effectively taught me medical management skills (Survey Group #2)	CHA/P	4.33 (0.49)	4	4 – 5
I felt the debrief was conducted in a safe environment (Survey Group #1)	CHA/P	4.72 (0.47)	5	4 – 5
	Nurse, ER Technician	4.94 (0.24)		
I felt the debrief was conducted in a safe environment (Survey Group #2)	CHA/P	4.42 (0.51)	4	4 – 5
I felt the debrief promoted reflection and team discussion (Survey Group #1)	CHA/P	4.81 (0.40)	5	4 – 5
	Nurse, ER Technician	4.94 (0.24)		
I felt the debrief promoted reflection and team discussion (Survey Group #2)	CHA/P	4.58 (0.51)	5	4 – 5
Group #1 Total	n = 28	4.75 (0.19)	5	3-5
Group #2 Total	n = 12	4.42 (0.14)	4.5	4-5
Total Combined	40	4.59 (0.23)	5	3-5

Feasibility outcomes

When asked dichotomous positive/negative feasibility questions, the CHA/Ps responded 100% positive to acceptability, demand, and practicality questions and 91% positive to the implementation question. The nurses and ER technicians responded 98% positive to acceptability, 100% demand, 79% practicality, and 94% implementation. The key informants responded 100% positively to acceptability, practicality, and implementation, and 95% positively to demand (Table 6).

**Table 6:** Survey responses to feasibility questions (Yes / No) CHA/P: Community Health Aide/Practitioners; ER: Emergency Room

Feasibility Domain	Participant	% YES	% NO
Acceptability	CHA/P	100	0
	Nurse, ER Technician	98	2
	Key informant	100	0
Demand	CHA/P	100	0
	Nurse, ER Technician	100	0
	Key informant	95	5
Practicality	CHA/P	100	0
	Nurse, ER Technician	79	21
	Key informant	100	0
Implementation	CHA/P	91	9
	Nurse, ER Technician	94	6
	Key informant	100	0

The following themes emerged upon qualitative analysis.

In the acceptability domain, themes emerged across all respondents that simulation-based active learning is a useful and well-received way to prepare for responding to emergencies. Educators also note that collaborating on simulation facilitation enhanced cross-institutional collegiality.

In the demand realm, themes expressed across all respondents that this type of education is needed to standardize medical care and improve teamwork/communication skills and healthcare worker confidence, which has the potential to improve patient outcomes/safety.

In the practicality realm, themes emerged from the key informants and educators that funding and physical space would be necessary to realize a program and scheduling would be a challenge for a workforce spanning a large rural geographic region. Participants expressed they would participate if it was supported and expected by their managers, if continuing education credits were allotted, and if it fits into the standard workday.

In the implementation realm, themes emerged from educators and some key informants that there would need to be broader institutional buy-in to enforce a program with an allotted local champion responsible for it, especially given the reality of high staff turnover in the region. While educators felt comfortable running simple task-training simulations and leading ACLS courses as they had done prior to this project, they expressed discomfort with facilitating and debriefing more complex medical scenarios after just one workshop and two days of direct observation and feedback.

All respondents suggested the possibility of utilizing the robust telecommunication network already in place in the region to overcome barriers to accessing a high-quality, sustainable SBCE. CHA/Ps overwhelmingly asked for simulations “in-situ” in the village and sub-regional clinics, where the bulk of their medical practice takes place. They note their daily experience with telemedicine, tele-education, and teleconferencing, and suggest the potential to offer this training via this established telecommunication system to overcome the acknowledged barriers of time, space, and resources. See Tables 7-8 for sample quotations.

**Table 7:** Example quotations: acceptability and demand CHA/P: Community Health Aide/Practitioner; SIM: Simulation

Participant surveys	Educator focus groups	Key informant semi-structured interviews
“Helps a CHA/P [prepare] when she deals with a real emergency. We don’t have emergencies every day in our clinics. It will also help a CHA/P to think fast or know what to do in an emergency especially with babies.” (05)	“I think it helps them learn better when they can actually practice hands-on. I think they can develop more confidence from doing hands-on learning, and I think they are bored to tears when we just talk at them.” (48)	“This is a project that I have wanted to put in place for some time, it will give great benefit to both new and seasoned Health Aides in their practice, in a safe environment that is conducive to learning and retention skills.” (32)
“Simulation helps teach in a non-punitive – non-grading method and builds confidence in skills and communication. This overall will improve patient outcomes.” (12)	“[SIM affects my teaching] Undoubtedly. It's hard, one if you’re studying adult education, adult learning, to imagine quality teaching without it. If you really want retention.” (49)	“We don’t currently have this program and I think with staff turnover and new staff starting all the time, this could be a valuable way to improve our patient care and safety.” (31)
“With so much turnover in staff, frequent simulations ensure competency of all and consistency in care and patient outcome[s].” (20)	“I am an educator and I believe it to be the most effective form of education.” (49)	“I think this concept has the potential to improve both patient care and patient safety.” (31)

**Table 8:** Example quotations: practicality and implementation

Participant surveys	Educator focus groups	Key informant semi-structured interviews
“if integrated into your workday [this is] easy because it’s expected and not extra effort.” (15)	“My pipe dream about doing them in the village, to get support for that.” (48)	“Low-fidelity would be easy to implement, high-fidelity would require more resources.” (31)
“I make time for training that teaches me and improves patient outcomes.” (20)	“I think [telesimulation] may be the only way we can do a mock-code type thing in a village setting. This would be worth investigating to see. I think if we tried it in one village, and they had a positive experience, then we could spread that and use that.” (48)	“I do not see a negative effect only that it will not be effective without total buy into the opportunity by organizations. The biggest challenge is maintaining quality to ensure that learners are engaged in effective manner. This quality would be ensuring appropriate management of simulation environment including equipment, scenarios and training for those who run the simulations. I believe this could be an amazing asset to improving our patient outcomes.” (33)
“Sustainable if this is supported by the training center.” (04)	“The problem is this is a very unique place. A very unique financial structure. The turnover is very high, and it is hard to want to invest in a very expensive program that requires consistent investment to keep it running when 30% of your staff are here for 3 months at a time. They may benefit tremendously from that training but then someone else will benefit, not us, once they leave. So that is a barrier.” (49)	“The challenges I see are in time, and possibly travel.” (33)

## Discussion

We were able to conduct a simulation program that served a unique and disparate group of participants who practice broad-spectrum “bush” medicine. Despite widespread acceptability and demand, the full integration of a practical and implementable SBCE program would require larger institutional buy-in, established champion(s), funding, and creativity in overcoming scheduling and access issues.

Our team prioritized the training of local educators in simulation facilitation and created opportunities to practice these newly acquired skills with on-the-ground mentorship in an effort to integrate the didactic method into the existing CHA/P training program. Integration is another feasibility domain, operationalized as the level of system change needed to integrate a new program or process into an existing infrastructure or program [18]. The local educators expressed insecurity in their facilitation skills after just one day of simulation training and only two subsequent mentored practice sessions. Interestingly, the simulation participants gave overwhelmingly positive feedback to these sessions, reflected in their Likert scale perceived effectiveness scores and open-ended survey responses. The discrepancy between local educator confidence and positive participant feedback should be further explored in order to create a feasible program.

Participants, key informants, and educators alike overwhelmingly brought up the possibility of harnessing the power of the established telecommunication network unique to this setting to overcome the perceived barriers to integrating an SBCE curriculum. One possible solution to enhancing the feasibility of an SBCE program for providers in this remote and resource-limited setting is leveraging the telecommunication system to introduce telesimulation. Telesimulation is a replicable, low-cost, and robust tool that optimizes learning and instructor training with simulation in resource-limited settings [22]. Tele-co-debriefing has been utilized in transcontinental settings for simulation faculty development and remotely facilitated simulation has been shown to be as effective as traditional, locally facilitated simulation [23]. Future projects might consider developing a telesimulation program. The program could utilize a network of simulationists from more resourced areas to help co-facilitate remotely, with the intention to improve access to high-quality CE while continuing to build capacity in the local simulation facilitators themselves. Remote co-facilitation may be one way to provide ongoing mentorship to the local newly trained educators in an effort to enhance their comfort in simulation facilitation. Future studies can focus on demonstrating impacts on learners, facilitators, clinical outcomes, and other quality improvement metrics.

This study is limited by a small convenience sample size. Given that we chose to conduct a feasibility study in a real-life, busy hospital with the actual participants that would ultimately be involved in the proposed SBCE, the authors felt this was necessary. However, we acknowledge the disadvantage that the convenience sample does not accurately represent the entire population of providers seeking CE in this broader geographic region. Thus, feasibility results might be skewed and may represent a barrier. We limited the evaluation of the simulation to learner perceptions of utility and did not directly evaluate the impact on knowledge, acquisition, or communication skills. Pre- and post-tests and/or videotaping with subsequent review are ways to enhance the evaluation piece of simulation in the future.

## Conclusions

SBCE was found to be acceptable and in demand. Local educators were trained and facilitated simulations that were well-received by participants. While we were able to implement this research project, we uncovered barriers to the true integration of this SBCE program moving forward. A suggested, innovative solution to overcome some feasibility barriers was telesimulation. Future initiatives might consider developing a telesimulation tool to integrate into the established telecommunication system in the region. Studies can build upon this assessment and consider ways to innovate simulation delivery while studying the impact on cost, provider competence, and, ultimately, patient outcomes. The authors herein conclude that a formal low-fidelity SBCE program may be feasible within this rural Alaska Native healthcare system if the acknowledged barriers are addressed creatively.
